# Mosaic variegated aneuploidy syndrome caused by a CEP57 mutation diagnosed by whole exome sequencing

**DOI:** 10.1002/ccr3.1655

**Published:** 2018-06-21

**Authors:** Diana S. Brightman, Sehar Ejaz, Andrew Dauber

**Affiliations:** ^1^ Genetic Counseling Program Division of Human Genetics Cincinnati Children's Hospital Medical Center Cincinnati OH USA; ^2^ Pediatric Endocrinology Department of Pediatrics Nassau University Medical Center East Meadow NY USA; ^3^ Cincinnati Center for Growth Disorders Division of Endocrinology Cincinnati Children's Hospital Medical Center Cincinnati OH USA

**Keywords:** CEP57, genetic testing, mosaic variegated aneuploidy syndrome, whole exome sequencing

## Abstract

This case highlights an important lesson for laboratory genetic testing. Geneticists and Genetic Counselors should be aware that although rare, mosaic variegated aneuploidy should be considered if mosaic aneuploidies are observed on karyotype, particularly in the context of short stature.

## 1 INTRODUCTION

Mosaic variegated aneuploidy (MVA) syndrome is a rare disorder presenting with short stature, intellectual disability, and mosaic aneuploidies. Herein, we report a patient with MVA diagnosed via whole exome sequencing after two normal karyotype results. MVA should be considered if mosaic aneuploidies are observed on karyotype.

Mosaic variegated aneuploidy (MVA) syndrome is a rare disorder characterized by mosaic aneuploidies of many chromosomes.^1^ The inheritance is autosomal recessive and caused by biallelic mutations in *TRIP13*,* BUB1B,* or *CEP57*, which establish the spindle assembly checkpoint, stabilize mitotic spindle attachment, and stabilize mitotic microtubule attachment, respectively.^1^, ^2^, ^3^, ^4^, ^5^, ^6^, ^7^ The phenotype of MVA is nonspecific and includes growth retardation, intrauterine growth restriction (IUGR), microcephaly, facial dysmorphia, and intellectual disability (ID).^1^, ^2^, ^3^, ^8^ The nonspecific phenotype can result in patients being underdiagnosed. Additionally, although mosaic aneuploidies should be observed on karyotype, they may be dismissed as an artifact.^9^ In this report, we describe a patient with MVA caused by biallelic mutations in *CEP57* who was undiagnosed until whole exome sequencing (WES).

## 2 CLINICAL REPORT

The patient was an 11‐year 6‐month‐old Pakistani female who presented for an initial endocrine evaluation of short stature. She was born at 38 weeks of gestation to consanguineous parents. She had past diagnoses of IUGR, failure to thrive, developmental delay, mild ID, acanthosis nigricans, and precocious puberty. A physician in Pakistan treated her with leuprolide depot, growth hormone, and metformin for precocious puberty, idiopathic short stature, and acanthosis nigricans, respectively. Her physical examination revealed a prominent forehead and nose, frontal bossing, triangular face, and mild rhizomelic shortening of upper limbs. Her height was 51 inches (<3rd percentile), weighed 76 lbs (19th percentile), and BMI of 76th percentile. High‐resolution karyotype revealed a 46, XX karyotype.

## 3 MOLECULAR ANALYSIS

**Figure 1:**
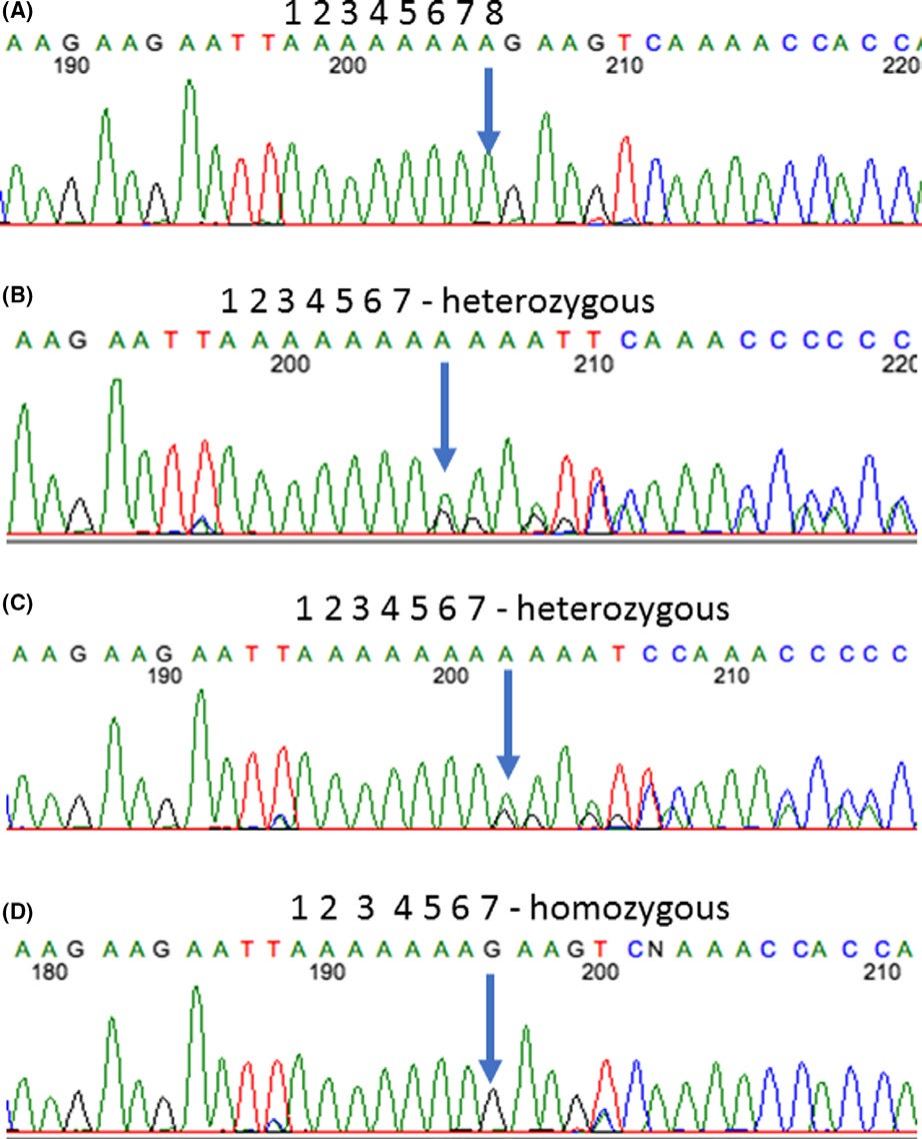
Sanger sequencing results confirm the *CEP57* frameshift mutation. Chromatograms of a control wild‐type sample (A) as well as the patient's mother (B), father (C), and the patient (D) confirm that the patient has a homozygous frameshift variant in *CEP57* (c.697delA, p.Lys235Argfs*31), while both of her parents are heterozygous for the same variant. The deleted nucleotide is indicated by an arrow

Informed consent was obtained from the parents and approved by the local institutional review board prior to molecular analysis. Based on the family history of consanguinity and unaffected parents, we suspected an autosomal recessive pattern of inheritance for which we performed WES. We hypothesized that the causal variant would be a rare, nonsynonymous, or frameshift homozygous variant. We excluded all variants with a minor allele frequency (MAF) >0.001 in the 1000 Genomes database (http://www.1000genomes.org) or in our internal exome database. There were 34 homozygous variants that fit these criteria. Review of each identified gene revealed only one that has previously been associated with growth retardation. This variant is a frameshift mutation in *CEP57* (c.697delA, p.Lys235Argfs*31) and is not listed in dbSNP or ClinVar but is present in the Exome Aggregation Consortium database (exac.broadinstitute.org) with a MAF of 2.576e^−5^. Truncating mutations in *CEP57* have been reported to cause MVA (OMIM 614114).^2^, ^3^ Sanger sequencing of *CEP57* in the patient and her parents confirmed that the patient had this homozygous frameshift mutation, while her parents each had a heterozygous frameshift mutation (Figure 1).

**Figure 2:**
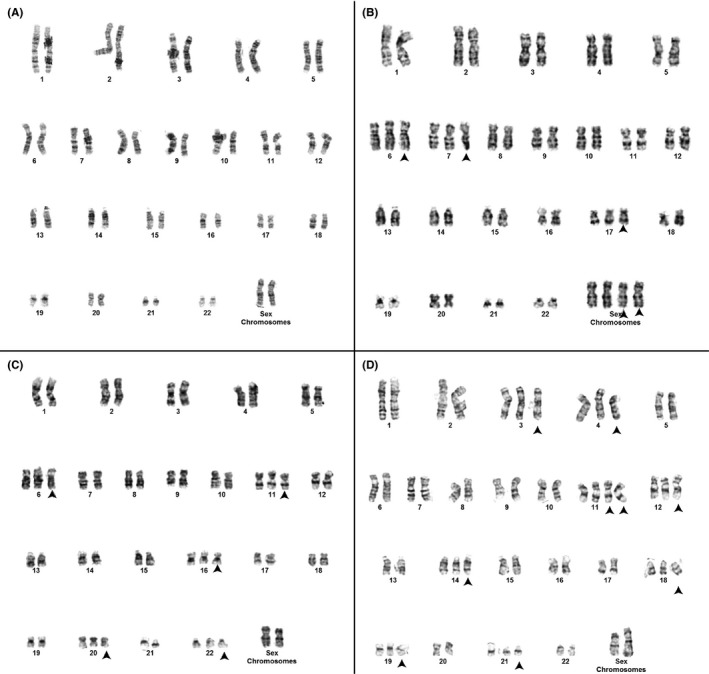
Chromosome results indicate mosaic variegated aneuploidy syndrome. The karyotype of 17 of 22 cells was 46,XX (A). Various aneuploidies were observed in the remaining five cells, three of which are pictured (B‐D). These include 51,XXXX,+6,+7,+17 (B), 51,XX,+6,+11,+16,+20,+22 (C), 55,XX,+3,+4,+11,+11,+12,+14,+18,+19,+21 (D). The aneuploidies are indicated with an arrowhead

Due to WES findings, we ordered a repeat karyotype from peripheral blood mononuclear cells. Surprisingly, the results from the clinical laboratory were normal 46,XX. After requesting a re‐review, 17 of 22 cells were 46,XX, while 5 cells had unique aneuploidies that the laboratory initially interpreted as artifacts (Figure 2). The mosaic aneuploidies confirmed the diagnosis of MVA.

## 4 DISCUSSION

Only 5 cases of MVA caused by *CEP57* mutations have been reported in the literature.^2^, ^3^ Interestingly, MVA caused by homozygous mutations in *BUB1B* or *TRIP13* is associated with an increased risk of cancer, but it is unknown if MVA caused by mutations in *CEP57* is associated with increased cancer risks.^1^, ^2^, ^3^, ^7^, ^10^ Had this been known, growth hormone might have not been prescribed. Consistent with prior reports of MVA caused by mutations in *CEP57*, this patient has growth retardation, IUGR, ID, rhizomelic limb shortening, and skull anomalies (temporal bossing and triangular face) (Table 1).^2^, ^3^ This patient also has precocious puberty and acanthosis nigricans, which may be previously undescribed features of MVA or may be unrelated to this syndrome. Additional case reports of patients with MVA caused by mutations in *CEP57* are needed to further define the phenotypic spectrum.

**Table 1:** Review of the phenotype of published cases of CEP57 mutations

	Previous reports	Present report	Total
Reference	Snape et al 2011, Pinson et al 2014	This Case Report	
Age	3 wk‐15 y	12 y 7 mo	3 wk‐15 y
Sex	3 Males, 2 Females	1 Female	3 Males, 3 Females
Ethnicity	Mexican, Caucasian, Moroccan	Pakistani	
Growth retardation	5/5	+	6/6
IUGR	3/5	+	4/6
Mild intellectual disability	2/4	+	3/4
Rhizomelic shortening	3/5	+	4/6
Skull anomalies	3/3	+	4/4
Precocious puberty	Not reported	+	
Acanthosis nigricans	Not reported	+	
Cancer	0/5	‐	0/6

IUGR, Intrauterine Growth Restriction.

This report highlights an important lesson for genetic testing. Karyotype was normal (46,XX) despite the presence of mosaic aneuploidies in a subset of cells. For a chromosome gain to be considered clonal, it must be observed in at least two cells per ISCN guidelines.^11^ This patient could have been diagnosed sooner had mosaic aneuploidies been detected and reported in her first karyotype. Laboratory and Clinical Geneticists should be aware that MVA should be considered if multiple mosaic aneuploidies are observed.

## AUTHOR CONTRIBUTIONS

DB: analyzed WES and wrote manuscript. SE: evaluated the patient, wrote the clinical description, and edited the manuscript. AD: analyzed WES, edited, and approved manuscript.

## CONFLICT OF INTEREST

The authors have no conflict of interests to disclose.

## References

[1] Hanks S , Coleman K , Reid S , et al. Constitutional aneuploidy and cancer predisposition caused by biallelic mutations in BUB1B. Nat Genet. 2004;36:1159‐1161. 15475955 10.1038/ng1449

[2] Snape K , Hanks S , Ruark E , et al. Mutations in CEP57 cause mosaic variegated aneuploidy syndrome. Nat Genet. 2011;43:527‐529. 21552266 10.1038/ng.822 PMC3508359

[3] Pinson L , Mannini L , Willems M , et al. CEP57 mutation in a girl with mosaic variegated aneuploidy syndrome. Am J Med Genet A. 2014;164A:177‐181. 24259107 10.1002/ajmg.a.36166

[4] He R , Wu Q , Zhou H , Huang N , Chen J , Teng J . Cep57 protein is required for cytokinesis by facilitating central spindle microtubule organization. J Biol Chem. 2013;288:14384‐14390. 23569207 10.1074/jbc.M112.441501 PMC3656293

[5] Chan GK , Jablonski SA , Sudakin V , Hittle JC , Yen TJ . Human BUBR1 is a mitotic checkpoint kinase that monitors CENP‐E functions at kinetochores and binds the cyclosome/APC. J Cell Biol. 1999;146:941‐954. 10477750 10.1083/jcb.146.5.941 PMC2169490

[6] Emanuele MJ , Stukenberg PT . Xenopus Cep57 is a novel kinetochore component involved in microtubule attachment. Cell. 2007;130:893‐905. 17803911 10.1016/j.cell.2007.07.023

[7] Yost S , de Wolf B , Hanks S , et al. Biallelic TRIP13 mutations predispose to Wilms tumor and chromosome missegregation. Nat Genet. 2017;49:1148‐1151. 28553959 10.1038/ng.3883 PMC5493194

[8] Garcia‐Castillo H , Vasquez‐Velasquez AI , Rivera H , Barros‐Nunez P . Clinical and genetic heterogeneity in patients with mosaic variegated aneuploidy: delineation of clinical subtypes. Am J Med Genet A. 2008;146A:1687‐1695. 18548531 10.1002/ajmg.a.32315

[9] Micale MA , Schran D , Emch S , Kurczynski TW , Rahman N , Van Dyke DL . Mosaic variegated aneuploidy without microcephaly: implications for cytogenetic diagnosis. Am J Med Genet A. 2007;143a:1890‐1893. 17632782 10.1002/ajmg.a.31848

[10] Rio Frio T , Lavoie J , Hamel N , et al. Homozygous BUB1B mutation and susceptibility to gastrointestinal neoplasia. N Engl J Med. 2010;363:2628‐2637. 21190457 10.1056/NEJMoa1006565

[11] McGowan‐Jordan JS , Simons A , Schmid M , International Standing Committee on Human Cytogenomic Nomenclature . ISCN: an international system for human cytogenomic nomenclature. New York: Karger; 2016.

